# Supplementary material for: scRepertoire 2: Enhanced and efficient toolkit for single-cell immune profiling
Source: PLoS Comput Biol. 2025 Jun 27;21(6):e1012760. doi: 10.1371/journal.pcbi.1012760 (PMC12204475; doi:10.1371/journal.pcbi.1012760)
Supplement: S1 Table — Check marks equate to supported functionality for single-cell sequences. PHYLO/SHM, Phylogenetic and somatic hypermutation. *Immunarch supports PHYLO/SHM for non-single-cell formats. **VDJVIEW has feature differentiation for BCRs versus TCRs, however no apparent method for calling BCR clonotypes for BCRs beyond strict sequence-based methods. (DOCX) [file pcbi.1012760.s001.docx]

| Tool/Package Name | Version | Platform | Vignettes /Examples | Multiformat Support | Cross tool Integration | scRNA-seq Interaction | TCR Support | BCR  Support | Summary Metrics | Clustering | Multimodal integration | Phylo/SHM analysis |
| --- | --- | --- | --- | --- | --- | --- | --- | --- | --- | --- | --- | --- |
| scRepertoire | v2.5.0 | R | ✔ | ✔ | ✔ | ✔ | ✔ | ✔ | ✔ | ✔ | ✔ |  |
| scRepertoire [1] | v1.11.0 | R |  |  |  | ✔ | ✔ | ✔ | ✔ | ✔ |  |  |
| Dandelion [2] | v0.5.4 | Python | ✔ | ✔ | ✔ | ✔ | ✔ | ✔ | ✔ | ✔ | ✔ |  |
| enclone [3] | Beta | Rust | ✔ |  |  |  | ✔ | ✔ | ✔ | ✔ | ✔ | ✔ |
| Immcantation [4] | v4.4.0 | Python, R | ✔ | ✔ | ✔ | ✔ | ✔ | ✔ | ✔ | ✔ |  | ✔ |
| Immunarch | v0.9.1 | R | ✔ | ✔ |  |  | ✔ | ✔ | ✔ | ✔ |  | * |
| Platypus [5] | v3.6.0 | R | ✔ | ✔ |  | ✔ | ✔ | ✔ | ✔ | ✔ |  | ✔ |
| Scirpy [6] | v0.22.0 | Python | ✔ | ✔ | ✔ | ✔ | ✔ | ✔ | ✔ | ✔ | ✔ | ✔ |
| VDJdive | V1.8.0 | R | ✔ |  |  | ✔ | ✔ |  | ✔ | ✔ |  |  |
| DJVDJ | V0.1.0 | R | ✔ |  |  | ✔ | ✔ | ✔ | ✔ | ✔ |  |  |
| VDJVIEW[7] | NA | R |  |  |  | ✔ | ✔ | ** | ✔ |  |  |  |

**References**

1. Borcherding N, Bormann NL, Kraus G. scRepertoire: An R-based toolkit for single-cell immune receptor analysis. F1000Research. 2020;9: 47. doi:10.12688/f1000research.22139.2

2. Suo C, Polanski K, Dann E, Lindeboom RGH, Vilarrasa-Blasi R, Vento-Tormo R, et al. Dandelion uses the single-cell adaptive immune receptor repertoire to explore lymphocyte developmental origins. Nat Biotechnol. 2024;42: 40–51. doi:10.1038/s41587-023-01734-7

3. Jaffe DB, Shahi P, Adams BA, Chrisman AM, Finnegan PM, Raman N, et al. enclone: precision clonotyping and analysis of immune receptors. bioRxiv; 2022. p. 2022.04.21.489084. doi:10.1101/2022.04.21.489084

4. Gupta NT, Vander Heiden JA, Uduman M, Gadala-Maria D, Yaari G, Kleinstein SH. Change-O: a toolkit for analyzing large-scale B cell immunoglobulin repertoire sequencing data. Bioinformatics. 2015;31: 3356–3358. doi:10.1093/bioinformatics/btv359

5. Yermanos A, Agrafiotis A, Kuhn R, Robbiani D, Yates J, Papadopoulou C, et al. Platypus: an open-access software for integrating lymphocyte single-cell immune repertoires with transcriptomes. NAR Genomics Bioinforma. 2021;3: lqab023. doi:10.1093/nargab/lqab023

6. Sturm G, Szabo T, Fotakis G, Haider M, Rieder D, Trajanoski Z, et al. Scirpy: a Scanpy extension for analyzing single-cell T-cell receptor-sequencing data. Bioinformatics. 2020;36: 4817–4818. doi:10.1093/bioinformatics/btaa611

7. Samir J, Rizzetto S, Gupta M, Luciani F. Exploring and analysing single cell multi-omics data with VDJView. BMC Med Genomics. 2020;13: 29. doi:10.1186/s12920-020-0696-z
