# Supplementary material for: scRepertoire 2: Enhanced and efficient toolkit for single-cell immune profiling
Source: PLoS Comput Biol. 2025 Jun 27;21(6):e1012760. doi: 10.1371/journal.pcbi.1012760 (PMC12204475; doi:10.1371/journal.pcbi.1012760)
Supplement: S2 Table — (DOCX) [file pcbi.1012760.s002.docx]

| **Exported Function** | **Change Type** | **Description** |
| --- | --- | --- |
| **combineBCR** | Enhanced | Improved performance using C++ and now permits inclusion of unproductive contig data with the new filterNonproductive parameter. |
| **combineTCR** | Enhanced | Improved performance using C++ and now permits inclusion of unproductive contig data with the new filterNonproductive parameter. |
| **loadContigs** | Enhanced | Expanded to support multiple formats, updated dual-chain support, automatic detection of format. |
| **clonalOverlap** | Fixed | Corrected an issue with the group.by parameter. |
| **vizGenes** | Fixed | Corrected an issue where IGH/K/L genes were being misidentified. |
| **getCirclize** | Fixed / Enhanced | Fixed denominator issue and later refactored to prevent assumptions with the new include.self argument . |
| **clonalCluster** | Fixed / Enhanced | Improved exportGraph and issues with null graphs |
| **annotateInvariant** | New | Introduced functionality to annotate invariant TCRs for mouse and human. |
| **quietTCRgenes** | New | Introduced to filter out known TCR gene signatures. |
| **quietBCRgenes** | New | Introduced to filter out known BCR gene signatures. |
| **quietVDJgenes** | New | Introduced to filter both TCR and BCR gene signatures simultaneously. |
| **getContigDoublets** | New | New Function to identify TCR/BCR doublets prior to the combineExpression() preprocessing step. |
| **percentVJ** | New | New function to calculate the percent of V and J pairings. |
| **percentAA** | New | New function to calculate percentage of amino acids. |
| **percentGenes** | New | New function to calculate the percentage of genes. |
| **percentKmer** | New | New function to calculate the percentage of kmers. |
| **exportClones** | New | Introduced to facilitate the export of clone data. |
| **positionalEntropy** | New | New metric for calculating positional entropy. |
| **positionalProperty** | New | New Function to calculate amino acid properties. |
| **clonalSizeDistribution** | Renamed | Renamed from clonotypeSizeDistribution. |
| **clonalScatter** | Renamed | Renamed from scatterClonotypes. |
| **highlightClones** | Renamed | Renamed from highlightClonotypes. |
| **clonalAbundance** | Renamed | Renamed from abundanceContig. |
| **alluvialClones** | Renamed | Renamed from alluvialClonotypes. |
| **clonalCompare** | Renamed / Enhanced | Renamed form compareClonotypes, allow for proportion and raw numbers, add new labeling and highlighting functionality, |
| **clonalLength** | Renamed / Fixed / Enhanced | Renamed from lengthContigs and improved single-chain and NA handling, |
| **clonalOccupy** | Renamed / Updated | Renamed from occupiedscRepertoire and rewritten to improve counting and NA handling |
| **clonalOverlay** | Updated | Modified argument names to include cutpoint and cut.category for selecting clonal proportions versus frequencies. |
| **StartracDiversity** | Updated | Re-implemented to eliminate the intermediary startrac-class object. |
| **powerTCR** | Updated | Now implemented locally to reduce external dependencies. |
| **clonalDiversity** | Updated | No longer automatically orders samples; the x.axis parameter is now separated from the group.by parameter. |
