# Supplementary material for: scRepertoire 2: Enhanced and efficient toolkit for single-cell immune profiling
Source: PLoS Comput Biol. 2025 Jun 27;21(6):e1012760. doi: 10.1371/journal.pcbi.1012760 (PMC12204475; doi:10.1371/journal.pcbi.1012760)
Supplement: S1 Text — (DOCX) [file pcbi.1012760.s006.docx]

**Methods**

*Benchmarking Analysis*

Computational performance was assessed for four widely used scAIRR-seq toolkits, vdjdj (v0.1.0), immunarch (v0.9.1), scRepertoire v1 (v1.11.0) and v2, by measuring both execution time and peak memory consumption across datasets of increasing scale. The data was derived from 2×10^4^ to 1×10^6^ cells that were randomly sampled from a cohort of 18670600 individual contigs and processed independently by each pipeline, with ten replicate runs per dataset size to account for run‐to‐run variability. Execution times were captured in seconds, and memory footprints in megabytes, using bench (v1.1.4) R package under identical hardware and software conditions (M1 Macbook Pro with 64 Gb LPDDR5-6400 Memory). For each pipeline and dataset size, we report the median of ten measurements with a 95% confidence interval. Benchmarking of scRepertoire v1 was discontinued after 256,000 cells due to memory consumption.

*Single-Cell Processing and Analysis*

Aligned single-cell RNA sequences were obtained from previously published datasets [1–3]. Sequencing runs were processed with Seurat (v5.2.1) [4], and filtering criteria were applied to retain only cells with ≥ 100 features, < 10% mitochondrial gene expression, and total counts below two standard deviations above the mean. Doublets were identified using scDblFinder (v1.18.0) [5] and removed; pure T cell populations were further ensured by applying scGate (v1.6.2) [6] and confirming the presence of TCR sequences with scRepertoire.

A total of 2,500 variable genes were calculated using the VST method with the manual removal of TCR and/or BCR VDJ genes. Gene expression values were normalized and scaled using percent of mitochondrial genes as latent variables, followed by principal component analysis was performed. To integrate individual sequencing runs, Harmony (v1.2.3) [7] was employed, and subsequent dimensionality reduction and clustering were performed on the first 30 Harmony-corrected dimensions. Clustering was executed using the Leiden algorithm at variable resolutions (0.4 for GSE169440, 0.6 for Paley et al., and 0.4 for Borcherding et al.), with adjustments made based on cell type and dataset size.

TCRs corresponding to YEIH^232-240^ were annotated by performing a grep search of the TRB amino acid sequence to include the following motifs SYST|TYST|TFST|YYST|GYST|LYST and verifying the presence of a TRAV21 gene in the corresponding ɑ-chain. Spike^167-180^ sequences were annotated according to previously published datasets from HLA-DPB1*04 individuals and tetramer-sorted sequences [3,8]. STARTRAC estimates were calculated in scRepertoire based on compartmental comparison between aqueous humor and blood samples [9]. Embedding of the TCRA sequence utilized the Atchley Factor CNN model in Trex [3]. Principal component analysis was performed using centered and scaled latent dimensions.

*Code Availability*

All code for the supplemental analyses are available at <https://github.com/> [BorchLab/scRepertoire.v2_manuscript](https://github.com/BorchLab/scRepertoire.v2_manuscript) within the quarto markdown file, SupplementalAnalysis.qmd.

**References**

1. Jiang R, Meng H, Raddassi K, Fleming I, Hoehn KB, Dardick KR, et al. Single-cell immunophenotyping of the skin lesion erythema migrans identifies IgM memory B cells. JCI Insight. 2021;6: e148035. doi:10.1172/jci.insight.148035

2. Paley MA, Yang X, Hassman LM, Penkava F, Garner LI, Paley GL, et al. Mucosal signatures of pathogenic T cells in HLA-B*27^+^ anterior uveitis and axial spondyloarthritis. JCI Insight. 2024;9. doi:10.1172/jci.insight.174776

3. Borcherding N, Kim W, Quinn M, Han F, Zhou JQ, Sturtz AJ, et al. CD4+ T cells exhibit distinct transcriptional phenotypes in the lymph nodes and blood following mRNA vaccination in humans. Nat Immunol. 2024;25: 1731–1741. doi:10.1038/s41590-024-01888-9

4. Hao Y, Stuart T, Kowalski MH, Choudhary S, Hoffman P, Hartman A, et al. Dictionary learning for integrative, multimodal and scalable single-cell analysis. Nat Biotechnol. 2024;42: 293–304. doi:10.1038/s41587-023-01767-y

5. Germain P-L, Lun A, Meixide CG, Macnair W, Robinson MD. Doublet identification in single-cell sequencing data using *scDblFinder*. F1000Research; 2022. doi:10.12688/f1000research.73600.2

6. Andreatta M, Berenstein AJ, Carmona SJ. scGate: marker-based purification of cell types from heterogeneous single-cell RNA-seq datasets. Bioinformatics. 2022;38: 2642–2644. doi:10.1093/bioinformatics/btac141

7. Korsunsky I, Millard N, Fan J, Slowikowski K, Zhang F, Wei K, et al. Fast, sensitive and accurate integration of single-cell data with Harmony. Nat Methods. 2019;16: 1289–1296. doi:10.1038/s41592-019-0619-0

8. Mudd PA, Minervina AA, Pogorelyy MV, Turner JS, Kim W, Kalaidina E, et al. SARS-CoV-2 mRNA vaccination elicits a robust and persistent T follicular helper cell response in humans. Cell. 2022;185: 603-613.e15. doi:10.1016/j.cell.2021.12.026

9. Zhang L, Yu X, Zheng L, Zhang Y, Li Y, Fang Q, et al. Lineage tracking reveals dynamic relationships of T cells in colorectal cancer. Nature. 2018;564: 268–272. doi:10.1038/s41586-018-0694-x
