# Supplementary material for: scRepertoire 2: Enhanced and efficient toolkit for single-cell immune profiling
Source: PLoS Comput Biol. 2025 Jun 27;21(6):e1012760. doi: 10.1371/journal.pcbi.1012760 (PMC12204475; doi:10.1371/journal.pcbi.1012760)
Supplement: Attachment — Submitted filename: scRepertoire2_reviewer_comments.docx [file pcbi.1012760.s008.docx]

**Review Comments**

**Reviewer #1: Exploring a fresh version of scRepertoire is a significant and valuable contribution. It is necessary to improve and expand the evaluation and presentation of results and performance.**

**The abstract is quite generic, so the authors could include summary statistics to assess the performance of their method and support their assertions in the abstract.**

We have updated the abstract to include an overall summary of performance comparing scRepertoire versions 1 and 2. In addition, we have benchmarked several other popular scAIRR-seq profiling toolkits, which are available in Supplemental Figure 1.

**Please explain the acronyms before starting to use them. For instance, what does 'scAIRR-seq' mean?**

We thank the reviewer for this comment. scAIRR-seq is defined in the first sentence of the abstract. However, we have reviewed the entire manuscript and added additional definitions of acronyms before using them, such as complementarity-determining region 3 (CDR3).

**You should add multiple references to support your statements. For instance, this sentence requires multiple references: "Many tools lack robust integration for immune receptor profiling with transcriptomic data or flexibility in data export formats, which hinders reproducibility and cross-platform compatibility in expanding datasets."**

We have added citations to the respective statement, including an extensive review of the current computational framework capabilities for scAIRR analyses. We have reviewed the manuscript and added additional citations where appropriate.

**It is necessary to create a table and compare the functionality of your package with those of the published packages, including scRepertoire v1.**

We thank the reviewer for this suggestion. We have added a Supplemental Table 1 that compares software that analyzes scAIRR-seq data at the post-alignment stage (comparable to scRepertoire). We have also added Supplemental Table 2, comprehensively summarizing the changes and additions to the scRepertoire v2.

**The aim of scAIRR-seq and Single Cell Immune Profiling is to provide comprehensive insights into the immune system at the single-cell level. However, their scope and integration of different data modalities have nuances. You need to discuss this in your article. Can your tool be used for Single Cell Immune Profiling?**

scRepertoire is a toolkit for single-cell immune profiling. This is clarified in the abstract, the introduction, and the data formats supported in the R environment (Seurat and SingleCellExperiment). We respect the reviewer's comment but are unsure of the exact nature of the intent and would happily revise and respond if clarified.

**Can your tool help improve the antigen specificity information of scAIRR-seq?**

Several functions in scRepertoire may may assist in the identification or understanding of antigen specificity - these include motif-based analyses provided by percentKmer() and closer examination of the CDR3 amino acid sequences using positionalProperty() or positionalEntropy(). In addition, the compatible Trex package has been used to identify novel spike-specific T follicular helper cells that were confirmed in vitro (Borcherding, et al Nature Immunology 2024) and Ibex has been used to identify HLA-specific B cell receptors in the transplant setting (manuscript under revision). Lastly, we are committed to inter-compatibility for alternative pipelines and support the interoperability of TCRmatch via the exportClones() function. We have added the following details to the manuscript.

**The method section focuses mainly on reviewing functions. It would be helpful if you could give more details about the methodology behind each function to help understand their functionality and as a guide for picking the appropriate one for downstream analysis. Additionally, it is important to highlight any new features in your package. Is it simply a collection of previously released tools or do you include additional components to address the shortcomings in the field?**

The software article format has a Design and Implementation section that we have used to focus on new features added to scRepertoire 2 from the previous version and have clarified this in the text. We have expanded this section to include additional details on methodology but are limited by the size of the article format. We have authored an extensive user guide on the pkgdown website that allows users to learn about the underlying methodology and for help in picking appropriate tools for downstream analysis:<https://www.borch.dev/uploads/screpertoire/>.

**The results section will become more extensive by presenting examples of how this new tool enhances biological interpretation. Can you provide a demonstration of biological insights that can be achieved using ScRepertoire?**

We elected to use the erythema migrans study as a demonstration of the novel feature for the scRepertoire v2 as it is one of the only public data sets with both TCR and BCR immune profiling and multiple tissues from the same individuals. However, the point is well taken that additional analysis asking focused questions for biological insights would improve the manuscript. We have added several such analyses as Supplemental Figure 2 - HLA-B27-associated autoimmune disease with defined pathogenic TCRs and Supplemental Figure 3 - HLA-DPB1*04 response to COVID-19 mRNA vaccination in the lymph node.

**I am unable to find a discussion section. Is it not necessary to include a discussion section?**

The manuscript is submitted as a software article and per the journal guidelines, does not have a discussion section.

**Reviewer #2: The manuscript presents scRepertoire 2, a major update to an R-based toolkit for single-cell adaptive immune receptor repertoire sequencing (scAIRR) analysis. The package optimizes computational efficiency, expands data format compatibility, introduces novel repertoire analysis features, and integrates with deep learning frameworks. A case study on erythema migrans lesion samples demonstrates its practical application, though validation across multiple disease contexts would strengthen the work.

Major Strengths
Substantial performance gains via C++ integration, reducing computational complexity from quadratic to linear scaling
Expanded data format support with automatic format detection, eliminating the need for file conversion
Novel analytical tools (positional entropy, amino acid property analysis, k-mer distributions) enable deeper repertoire characterization
Enhanced visualization modules and improved metrics for repertoire diversity analysis
Seamless integration with machine learning frameworks supports predictive modeling in computational immunology
The erythema migrans case study effectively demonstrates the package's capabilities**

**Major Weaknesses**
**No benchmarking against competing tools to quantitatively demonstrate efficiency gains
Limited validation based on a single dataset—additional disease models would improve generalizability**
**Insufficient technical details on algorithmic implementations, particularly for novel features
Lack of error case analysis—no discussion of potential failure scenarios
Minimal validation of new metrics across diverse datasets**

**Recommendations**
**Include benchmarking results comparing performance against tools like Immcantation, scirpy, and TRUST4**

We thank the reviewer for this suggestion. We have benchmarked several other popular scAIRR-seq profiling toolkits (immunarch and djvdj) in addition to scRepertoire v1 and they are available in Supplemental Figure 1. We have updated the text in the manuscript to refer to these benchmark results.

**Expand dataset validation using scAIRR data from cancer immunotherapy, autoimmune diseases, or vaccine studies**

Although we agree in principle with the reviewer that more extensive analysis across biological contexts would add strength to the manuscript, we are unfortunately limited to the short format of the software article. We have added additional supplemental analyses (Supplemental Figure 2 and Supplemental Figure 3) that focus on direct biological questions using studies of autoimmunity and vaccination, with reference in the main article, and have included all the code for these supplemental analyses in the GitHub repo to assist future users.

**Provide detailed algorithmic descriptions for novel features**

We have expanded the Design and Implementation section to add additional details based on the new features. However, we are limited by the space of the Software Article format in providing the additional information. Extensive details are provided on the pkgdown website corresponding to scRepertoire:<https://www.borch.dev/uploads/screpertoire/>.

**Discuss potential failure cases (e.g., low cell counts, dropout effects, misaligned assignments)**

scRepertoire functions on aligned sequences, the examples provided by the reviewer an issue for the preprocessing and alignment workflows of the formats themselves. We have added this as a caveat in the data compatibility section.

**Enhance comparison with alternative tools to contextualize unique contributions**

**Include runtime efficiency comparisons on large-scale datasets**

As previously indicated, we have added this to the manuscript in Supplemental Figure 1.

**Explore future directions such as spatial transcriptomics integration and multi-omics approaches**

We agree with the reviewer; we have expanded the discussion of spatial transcriptomics and multi-omic integration and the possible implications of the immune response analysis.

**The manuscript makes a significant contribution to single-cell immunogenomics. The performance improvements, expanded analytical capabilities, and deep learning integration are valuable for immune profiling research. However, addressing the benchmarking gaps, dataset limitations, and validation concerns would substantially strengthen the work.**

**Reviewer #3: In the manuscript of Yang et al, the authors present an updated version of the single-cell immune repertoire analysis tool, scRepertoire2, with a strong emphasis on integrating diverse single-cell data formats for upstream processing and incorporating various amino acid autoencoders in the downstream analyses. In the updated package, the enhancements in immune repertoire summarization, with the focus on amino acid composition and gene usage, have the potential to drive further developments in the field. Overall, the manuscript is well-constructed and I recommend acceptance of the manuscript after minor revisions.**

**1. Authors demonstrate the updated scRepertoire2 by analyzing transcriptomic and immune repertoire profiling data from erythema lesions. Authors did a good job in detailing the new features. More quantitative benchmarking with other packages would help highlight the unique advantages of scRepertoire2.**

We agree with this reviewer’s comment, which was a common suggestion among all reviewers. We have benchmarked several other popular scAIRR-seq profiling toolkits (immunarch and djvdj) in addition to scRepertoire v1 and they are available in Supplemental Figure 1. We have updated the text in the manuscript to refer to these benchmark results.

**2. One unique improvement of scRepertoire 2 is this compatibility with ML packages (like Trex, Ibex and ImmApex). Although the authors have included vignettes in the online tutorials, it would be helpful is author could include a more detailed example in the manuscript, demonstrating how scRepertoire 2 can integrate with those packages and what unique biological insights can be acquired.**

We have added the application of the ML packages, namely TCR autoencoding, to Supplemental Figure 3, examining HLA-DPB1*04-restricted vaccination response. In addition to the main text, we have more clearly stated the biological insights that can be acquired from the application of these methods. Currently, we have the Ibex (BCR) story under revision and are preparing the immApex manuscript, which will provide more insight beyond the scope of this paper.

**3. Single-cell dataset volumes are increasing dramatically. For example, ParseBio has published several immune profiling datasets with over 1 million cells. Are there specific optimizations for large-scale dataset(like disk storage or memory management) implemented in this version? How does scRepertoire2 compatilble with large-scale data (>1 miilions cells)? Authors should provide the information on scalability and performance with very large datasets.**

Within the new benchmarking results of Supplemental Figure 1, for scRepertoire v2, the median processing time for 1 million cells was 81 seconds with a memory footprint of 9.96 gigabytes. This was a substantially better performance than version 1, which exceeded memory allocation limits and could not be tested using 1 million cells. Still, we estimate, based on the linear nature, would approximate 3 hours of processing time and over a terabyte of memory.

**4. In the future direction section, authors should discuss alternative approaches currently addressing similar problems and how further development of scRepertoire2 would fit within this landscape.**

In the manuscript, we added additional details on the alternative approaches for single-cell immune receptor analyses and antigen prediction. In addition, we have added more citations earlier in the manuscript on alternative software and a comprehensive comparison of different workflows in Supplemental Table 1. We plan to make scRepertoire more compatible with these workflows and have already worked with the creators of Dandelion to create an R implementation that allows for VDJ-space trajectory analysis on single-cell objects processed with scRepertoire.

**5. Please ensure all figures have clear legends and all abbreviations should be well-defined.**

We have also reviewed the manuscript to ensure all abbreviations and legends are clear/well-defined.

**Reviewer #4: The study by Yang et. al. presents scRepertoire 2, a computational R package designed for immune repertoire analysis. The tool integrates single-cell RNA-seq and TCR/BCR sequencing data, enabling users to characterize immune clonotypes in their transcriptomic context. The key strengths of this work include:**

**- Enhanced usability & accessibility: The redesign introduces intuitive function names, improved documentation, and a pkgdown website to support users.
- Expanded data compatibility: Supports multiple sequencing pipelines (e.g., 10x Genomics, AIRR, MiXCR, TRUST4), improving adaptability.
- Performance optimizations: Efficient C++ integration reduces computational complexity, allowing linear scalability for key functions.
- Comprehensive repertoire summarization: Advanced features such as entropy analysis, positional amino acid property mapping, and k-mer analysis enhance functional interpretation.
- Machine learning (ML) applications: Integration with Trex, Ibex, and ImmApex expands predictive modeling capabilities, particularly for immunology-related deep learning applications.

Overall, scRepertoire 2 is a well-designed computational tool for immune repertoire analysis, introducing significant enhancements in data compatibility, performance, and machine learning applications. However, critical aspects such as comparative benchmarking, validation of functional predictions, batch effect correction, and scalability testing remain underexplored. Addressing these limitations will further strengthen its utility in high-throughput immunogenomics.

I have some listed concerns & suggested improvements**

**1. Design and Implementation / Workflow:
The workflow efficiently integrates immune repertoire data, but it lacks explicit benchmarking against existing tools such as Immunarch, scIR, or TCRgrapher to demonstrate comparative performance improvements.
- Suggested Change: Include quantitative benchmarking (e.g., accuracy of clonal assignments, runtime comparisons).**

We have included Supplemental Figure 1 as a summary of the tools for scAIRR-seq in terms of the popular scAIRR-seq toolkit performance. We have focused on the most popular alternatives (immunarch and djvdj). As a note to the reviewer regarding their suggested pipelines, although TCRgrapher is especially intriguing in connecting multiple pipelines, it does not appear to perform single-cell immune profiling with two chains. In addition, scIR appears defunct, and the GitHub repo is nonexistent.

**2. Expanded Data Compatibility:
The newly introduced `loadContigs()` function automatically detects formats, which is a strong feature. However, the details of how misclassified formats are handled are not described.
- Suggested Change: Provide error-handling mechanisms (e.g., warnings for ambiguous format detection).**

We thank the reviewer for this comment and have improved the loadContigs() function. In the revised implementation of loadContigs(), auto-detection of the format is integrated with robust error handling. When the function is run in auto mode with directory input, it scans file name patterns to detect potential formats. If multiple candidate formats are found, the function warns about ambiguous detection and defaults to the first candidate, ensuring that the user is informed about misclassification. In cases where no matching files are found, the function also warns the user and returns an empty list. For pre-loaded data, the function inspects the column names to infer the format and will stop with an error if auto-detection fails. Users can assert the format if preferred, providing full control over the detection process when needed.

**3. Performance Optimizations:
While C++ acceleration improves runtime efficiency, the scalability for large repertoires (>1M cells) is not explicitly tested.
- Suggested Change: Include real-world benchmarking for large single-cell datasets, preferably using public datasets from immune cell atlases.**
As mentioned above, in the revision, we provide benchmarking data on the speed and memory improvement for scRepertoire versions 1 and 2. For the latter, the median processing time for 1 million cells was 81 seconds with a memory footprint of 9.96 gigabytes. This was a substantially better performance than version 1, which exceeded memory allocation limits and could not be tested using 1 million cells. Still, we estimate, based on the linear nature, would approximate 3 hours of processing time and over a terabyte of memory.

**4. Clonal Diversity Analysis:
While rarefaction analysis is robust, it lacks a discussion on statistical variability (e.g., how confidence intervals are derived).
-Suggested Change: Report statistical uncertainties (e.g., confidence intervals, bootstrap variability).**

Thank you for your valuable comment. We have updated the manuscript and function documentation to explicitly detail that confidence intervals are derived via bootstrap resampling, thereby quantifying the statistical variability of the diversity estimates.

**5. ML Applications:
The ML component is promising, but it lacks clarity on training data availability and whether models are trained on independent datasets.
- Suggested Change: Provide details on model generalizability, including cross-validation performance on independent cohorts.**

The machine learning and deep learning capabilities of the companion packages - Trex, Ibex, immApex - are difficult to address in a software article focused on scRepertoire. Two of the packages - Ibex (in revision) and immApex (in preparation) will include additional details on model generation and best practices for immune receptor-based algorithms. We have clarified in the text these packages are separate and distinct from scRepertoire and have used Trex to perform a portion of the analysis in Supplemental Figure 3. More details on the Trex algorithm are available in the original publication by Borcherding et al. Nature Immunology 2024.
